# Secondary prevention of heart disease – knowledge among cardiologists and Ω-3 (Omega-3) fatty acid prescribing behaviors in Karachi, Pakistan

**DOI:** 10.1186/1471-2261-9-4

**Published:** 2009-01-27

**Authors:** Saqib A Gowani, Sana Shoukat, Ather M Taqui, Hashim M Hanif, Wasiq F Ravasia, Zeeshan Qadri, Sajid H Dhakam

**Affiliations:** 1 Section of Cardiology, Department of Medicine, Aga Khan University, Aga Khan, Pakistan

## Abstract

**Background:**

The use of omega-3 fatty acids is a currently proven strategy for secondary prevention of heart disease. The prescription practices for this important nutraceutical is not currently known. It is imperative to assess the knowledge of cardiologists regarding the benefits of omega-3 fatty acids and to determine the frequency of its prescription. The aim of the study was to determine the practices and associations of dietary fish prescribing among cardiologists of Karachi and to assess their knowledge of fish oil supplementation and attitudes toward dietary practices.

**Methods:**

A cross sectional survey was conducted during the period of January to March, 2008. A self report questionnaire was employed. All practicing cardiologists of Karachi were included in the study. Multiple logistic regression analysis was performed to determine the independent factors associated with high fish prescribers.

**Results:**

The sample comprised of a total of 163 cardiologists practicing in Karachi, Pakistan. Most (73.6%) of the cardiologists fell in the age range of 28 – 45 years and were male (90.8%). High fish prescribers only comprised 36.2% of the respondents. After adjusting for age and gender, multivariate analysis revealed that only the variable of knowledge about fish oil's role in reducing sudden cardiac death was independently associated with high fish prescribers OR = 6.38 [95% CI 2.58–15.78].

**Conclusion:**

The level of knowledge about the benefits of omega-3 fatty acids is high and the cardiologists harbor a favorable attitude towards dispensing dietary fish advice. However, the prescription practices are less than optimal and not concordant with recommendations of organisations such as the American Heart Association and National Heart Foundation of Australia. The knowledge of prevention of sudden cardiac death in CVD patients has been identified as an important predictor of high fish prescription. This particular life-saving property of omega-3 fatty acids should be the focus of any implemented educational strategy targeted to improve secondary CVD prevention via omega-3 fatty acid supplementation.

## Background

The burden of non communicable diseases is adding on to that of communicable illnesses in the developing countries and in fact, rising to alarming levels[1]. The South Asian region houses greater than 20% of the world's population and cardiovascular disease (CVD) is a leading cause of morbidity, mortality and disability in this region[2]. Individuals acquire heart disease ten years earlier than the average in the west and hence lose considerable productive years of working life[3]. Pakistan is the sixth largest country in the world with a population of 169 million[4] and evidence suggests that every one in four middle aged adults has prevalent coronary artery disease[5].

Major efforts now are being directed towards prevention rather than treatment of cardiovascular disease via primary and secondary prevention[6]. One evidence-based strategy is to increase amount of omega 3 fatty acids, mainly docosahexaenoic (DHA) and eicosapentaenoic acids (EPA), in the diet. The American Heart Association has recommended 1 gram of combined EPA and DHA for patients with coronary disease[7,8]. The benefit of the use of omega 3 fatty acids in diet and as supplements in secondary prevention of cardiovascular disease is supported by a large body of evidence which includes randomized clinical trials [9-11], meta-analysis[12] and systematic review[13]. Two large randomized clinical trials demonstrated that treatment with n-3 polyunsaturated fatty acids (PUFAs) significantly lowered the risk of cardiovascular death, non-fatal myocardial infarction, and stroke in post myocardial infarction patients[9,10]. An Italian trial showed that the ingestion of 1 g/day of n-3 PUFAs resulted in the reduction of total mortality, most of which was accounted by a reduction in sudden cardiac death. In fact, assessment of the omega-3 index – a measure of the concentration of EPA and DHA in erythrocytes is recommended, with a higher index reducing the likelihood of sudden cardiac death by 90%[14]. In lieu of the above evidence, international cardiac societies recommend the intake of 1 g/day of the two above mentioned omega-3 fatty acids for treatment after a myocardial infarction, prevention of sudden death, and secondary prevention of cardiovascular disease [15-17].

The use of omega-3 fatty acids as part of the diet has multiple benefits and it has been encouraged among primary care providers to prescribe where needed[18]. There is a paucity of literature on the knowledge, attitudes and prescribing patterns of physicians regarding omega-3 fatty acids. One study conducted on primary care physicians showed that despite adequate knowledge regarding the benefits of use of omega-3 fatty acids, primary care physicians recommend its use for their CVD patients infrequently[19].

Pakistan is a developing country with poor health indicators. There is a weak primary health care infrastructure with no referral system in place. The cost of the health care is borne by the patients themselves. Awareness about the importance of prevention is poor and it is not uncommon to find patients seeking medical help only after a cardiovascular event has already occurred. Due to the absence of a referral system, a large number of patients will approach the specialists directly [20]. Cardiologists have an important role in effecting secondary prevention strategies for their patients like advising the use of omega-3 fatty acids. Moreover, cardiologists have the responsibility to lead educational and awareness campaigns for the primary care providers.

It is imperative to assess the knowledge of cardiologists regarding the benefits of omega-3 fatty acids and to determine the frequency of omega-3 acid prescription. This has not been explored before. The present study was undertaken to determine the practices and associations of dietary fish prescribing among cardiologists of Karachi and to assess their knowledge of fish oil supplementation and attitudes toward dietary practices.

## Methods

### Study type and study sample

This cross sectional survey was conducted during the period of January to March, 2008. All practicing cardiologists of Karachi were included in the study. Contact information of the participants was derived from the Pakistan Cardiac Society listing, drug representatives of all pharmaceutical companies working in Karachi and all hospitals listed in yellow pages, Karachi. The participants were delivered the questionnaire personally by data collectors who also collected them back personally in a week's time. Informed consent was taken and strict confidentiality was ensured. The study was conducted in compliance with the 'Ethical principles for medical research involving human subjects' of the Helsinki Declaration.

"Cardiologist" was defined as a physician who sees primarily cardiac patients regardless of post-graduate qualifications, the rationale being the fact that these are the actual cardiology service providers in the city. All practicing Cardiologists of the study had their medical schools as well as training with English Language as the medium as is the case in Pakistan. The anticipated sample size was approximately 180 cardiologists.

### Survey instrument

The questionnaire was adopted from Oh et. al with the authors permission[19]. The questionnaire was in the English language. Pretesting was carried out among the cardiologists of the study institution. These were not approached during data collection. The pretest sample comprised of 15 respondents. A few amendments were made and the questionnaire was finalized.

The questionnaire contained a total of 23 items that asked about physician practices, knowledge, and attitudes of dietary fish supplementation for patients with known CVD. Prescribing practices were described using interval categories ranging from almost always (> 80% of the time), often (60% to 80%), sometimes (40% to 59%), not often (20% to 39%) to almost never (< 20%). Case scenarios were added to categorize physician-prescribing practices further. Knowledge regarding fish oil's effects on triglycerides, secondary prevention of cardiovascular disease and sudden death was assessed. Additionally, questions assessing attitudes toward dietary prescribing were included. Responses were based on a 5-point Likert scale ranging from "strongly disagree" to "strongly agree". Finally, questions regarding the number of patients with cardiovascular disease seen per week, nutritional training, general demographics and source of information along with access to medical literature and scientific meetings were included. [See Additional file 1]

### Data Analysis

Dietary prescribing frequency was collapsed into 3 categories: high-prescribers (> 60%); moderate prescribers (40% to 59%); and low prescribers (< 40%). For the purpose of bivariate analysis, the cardiologists were split into two groups according to their fish prescription practices. The cardiologists who self-reported as high prescribers of dietary fish advice and additionally prescribed a fish diet to the hypothetical CVD patient in Q4 (see questionnaire in additional file) were classified as high fish prescribers. Responses to dietary knowledge questions were dichotomized into correct or incorrect.

The data was entered in Epi Data version 3.1 and analyzed in Statistical Package for Social Sciences 15.0 (SPSS, Inc., Chicago, IL, USA). Descriptive statistics were performed for physician demographics, dietary practices, knowledge and attitudes. The results were recorded as frequencies, means ± standard deviations (SD) and p-values. Tables and figures were used for comprehensive viewing of the results.

Bivariate comparison of variables was done between the high fish prescriber and low fish prescriber group. The Chi-square test and Fisher's exact test were employed for categorical variables, the Mann-Whitney *U *test for ordinal variables and the student's *t*-test for independent variables. The variables which had a p-value < 0.25 on bivariate analysis were subjected to a stepwise multiple logistic regression analysis to determine which variables were independent predictors of high fish prescription practice. Each of nutrition training, attitude, and knowledge were tested as a separate group to assess their contribution to variation in high fish prescribing. The Hosmer and Lemeshow goodness-of-fit test[21] was also used to determine the final model. All values were 2-tailed and a p-value of < 0.05 was taken as the criteria of significance for all purposes.

## Results

A total of 201 questionnaires were distributed and 178 cardiologists returned the form forms (Response rate = 88.6%). Fifteen forms were excluded because key variables were missing from them. One hundred and sixty three questionnaires comprised the dataset for analysis.

### Demographics

Our sample comprised of a total of 163 cardiologists practicing in Karachi, Pakistan. Table 1 outlines the demographics of the respondents. Most (73.6%) of the cardiologists fell in the age range of 28 – 45 years. The majority (90.8%) was male and 98.1% had received their residency training in Pakistan. About half (46.6%) of the cardiologists had a hospital-based primary practice and a quarter of them were either full-time or part-time faculty at an academic institution.

**Table 1:** Demographics and prescribing practices of the cardiologists

	**n = 163** **Mean ± SD or n (%)**
**Age **(years)	37.4 ± 8.4

**Gender**	
Male	148 (90.8)
Female	15 (9.2)

**Primary practice**	
Office based	87 (53.4)
Hospital based	76 (46.6)

CVD* patients seen per week	
1 to 10	18 (11.0)
11 to 20	26 (16.0)
21 to 30	58 (35.6)
31 to 40	29 (17.8)
> 40	32 (19.6)

**Nutrition training^†^**	
Medical School	128 (78.5)
Residency	38 (23.3)
Other	23 (14.1)
Any	154 (94.5)
None	9 (5.5)

**Prescriber of dietary advice^‡^**	
Low (< 40%)	0
Moderate (40% to 60%)	35 (21.5)
High (> 60%)	128 (78.5)

**Prescriber of fish advice^‡^**	
Low (< 40%)	19 (11.7)
Moderate (40% to 60%)	43 (26.4)
High (> 60%)	101 (62)

* CVD = Cardiovascular disease

^† ^Percentages will not add up to 100%.

^‡ ^Represents the frequency of advice given by the cardiologists to patients with known CVD.

### Prescribing practices

The majority (78.5%) of the cardiologists was high prescribers of general dietary advice to patients with known CVD. One hundred and one (62%) self-reported as high prescribers of fish advice. However, in response to Q4, 42 (41.6%) of these did not prescribe fatty fish diet to the hypothetical patient post myocardial infarction. Therefore, according to the criteria defined for high fish prescribers, only 36.2% of the 163 respondents qualified. In response to Q5b, where a similar patient specifically requests advice fish supplementation, 92.6% recommended or strongly recommended it.

### Dietary Knowledge and Dietary Attitudes

Table 2 shows the dietary knowledge and attitudes of the respondents. The cardiologists scored high on two of the questions regarding the general diet but their knowledge regarding the effect of antioxidants on cardiovascular mortality was markedly low. Only 5.5% of respondents answered it correctly. Regarding the questions on dietary knowledge specific to fish diet, the respondents scored moderately high.

**Table 2:** Dietary Knowledge and attitude of the cardiologists

DIETARY KNOWLEDGE*	**Number answering correctly (%)**
**Questions pertaining to general diet**	
Fruits and vegetables lower blood pressure	99 (60.7)
Antioxidants do not reduce cardiovascular mortality	9 (5.5)
Low sodium diets lower blood pressure	141 (86.5)
**Questions pertaining to fish diet**	
Fish oil reduces cardiovascular mortality	117 (71.8)
Fish oil reduces triglycerides	103 (63.2)
Fish oil reduces sudden cardiac death	106 (65)

**DIETARY ATTITUDES**	**Agree or strongly agree (%)**

Nutrition has an important part to play in the prevention of CVD^†^	153 (93.9)
The cardiologist has an essential role in giving dietary advice	149 (91.4)
The cardiologist has insufficient time to advise patients adequately	57 (35)
Advice given will impact on what people eat	139 (95.3)
Advice given will be effective in reducing CVD	148 (90.8)

* Statements represent correct answers to knowledge questions

^† ^CVD = Cardiovascular disease

Attitudes towards dietary advice in CVD are shown in Table 2. They were overall strongly positive.

### Bivariate analysis

Associations between high fish prescribers and the relevant variables are presented in Table 3. Regarding the questions on general dietary advice, the cardiologists who knew that fruits and vegetables lower blood pressure were significantly more likely to be high fish prescribers (p value = 0.006). There was no significant association found for the other two questions regarding general dietary advice. On the other hand, the cardiologists who correctly answered the three questions regarding fish diet advice were significantly more likely to be high fish prescribers (p values < 0.05, see Table 3 for individual p values).

**Table 3:** Associations between High Fish Prescribers and Dietary Knowledge, Training, and Attitudies variables.

**Variables**	**High Fish Prescribers**		**p value**
		
	**No** **N = 104**	**Yes** **n = 59**	
**Demographics**			

Age (years)	36.3 ± 8.9	39.4 ± 7.2	0.023
Male gender	91 (87.5)	57 (96.6)	0.087

**Dietary Knowledge**			

**Questions pertaining to general diet**			
Fruits and vegetables lower blood pressure	55 (52.9)	44 (74.6)	0.006
Antioxidants do not reduce cardiovascular mortality	7 (6.7)	2 (3.4)	0.490
Low sodium diets lower blood pressure	87 (83.7)	54 (91.5)	0.158
**Questions pertaining to fish diet**			
			
Fish oil reduces cardiovascular mortality	69 (66.3)	48 (81.4)	0.041
Fish oil reduces triglycerides	57 (54.8)	46 (78.0)	0.003
Fish oil reduces sudden cardiac death	54 (51.9)	52 (88.1)	< 0.001

**Nutrition Training**			

Medical School	75 (72.1)	53 (89.8)	0.008
Residency	31 (29.8)	7 (11.9)	0.009
Other	16 (15.4)	7 (11.9)	0.535
Any	96 (92.3)	58 (98.3)	0.158

**Dietary Attitudes**			

Nutrition has an important part to play in the prevention of CVD			
Strongly disagree	2 (1.9)	--	0.667
Disagree	1 (1.0)	--	
Neutral	5 (4.8)	2 (3.4)	
Agree	48 (46.2)	29 (49.2)	
Strongly agree	48 (46.2)	28 (47.5)	

The cardiologist has an essential role in giving dietary advice			
Strongly disagree	--	--	0.270
Disagree	--	--	
Neutral	11 (10.6)	3 (5.1)	
Agree	54 (51.9)	30 (50.8)	
Strongly agree	39 (37.5)	26 (44.1)	

The cardiologist has insufficient time to advise patients adequately			
Strongly disagree	18 (17.3)	11 (18.6)	0.590
Disagree	14 (13.5)	4 (6.8)	
Neutral	32 (30.8)	27 (45.8)	
Agree	30 (28.8)	13 (22.0)	
Strongly agree	10 (9.6)	4 (6.8)	

Advice given will impact on what people eat			
Strongly disagree	1 (1.0)	--	0.092
Disagree	4 (3.8)	2 (3.4)	
Neutral	15 (14.4)	2 (3.4)	
Agree	60 (57.7)	38 (64.4)	
Strongly agree	24 (23.1)	17 (28.8)	

Advice given will be effective in reducing CVD			
Strongly disagree	--	--	0.881
Disagree	3 (2.9)	--	
Neutral	6 (5.8)	6 (10.2)	
Agree	62 (59.6)	33 (59.0)	
Strongly agree	33 (31.7)	20 (33.9)	

The Pearson's chi square test, Fisher's exact test and Mann-Whitney U test was used for categorical variables as appropriate and the student's *t*-test was used for continuous variables.

The majority of cardiologists had received nutrition training in medical school. Few had received it during residency. High fish prescribers were significantly more likely to have received nutrition training in medical school (p value = 0.008). However, this was converse for nutrition training during residency.

There were no significant differences seen in attitudes towards dietary advice in CVD between high fish prescribers and the rest of the respondents.

### Multivariate analysis

The variables with a p value of < 0.25 on bivariate analysis were entered in a stepwise multiple logistic regression analysis model (See Additional file 2). The first model included the following variables: demographics, general dietary and fish knowledge variables, dietary attitudes and nutrition training. Subsequently, reduced models were obtained to determine independent factors associated with high fish prescribers. At each step, the factors which did not contribute independent statistical information were removed. The multiple regression analysis revealed that the knowledge of the effect of fish oil on sudden cardiac death was significantly associated with high fish prescribing behaviors in all models. The final model, independent of age and gender, showed the odds ratio of this knowledge variable to be 6.38 with 95% CI of 2.58 to 15.78.

## Discussion

The key finding of this city-wide survey is a discrepancy in knowledge, attitudes and practice by cardiologists as shown by the low level of prescription of omega-3 fatty acids as supplement or in diet to CVD patients despite possessing adequate knowledge regarding its benefits and a self-reported strongly positive attitude towards fish prescription for this patient population. This is concordant with Oh and colleagues who conducted a similar study assessing knowledge, attitude and fish prescribing patterns among Washington State primary care physicians[19]. Knowledge regarding the use of omega 3 fatty acid in prevention of sudden cardiac death was independently associated with higher fish prescribing; consistent with results of Oh et al [19].

The present study found an overall high degree of knowledge as well as prescription of general dietary advice among the respondents. The vital role of health professionals' dietary advice in reducing the risk of cardiovascular disease has been supported by the literature[22,23] and hence it is comforting to know that the local cardiologists have reported strong knowledge, a positive attitude and a high prescription of this important measure. When compared to the percentage of physicians with self-reported high general dietary advice prescription (78.5%), prescription of fish advice was lower (62%). And when the prescription of fish advice was further assessed by specific scenario question, the number reduced by about half (36.2%). This is similar to Oh and colleagues' findings where a significant disparity was noted between perceived high fish prescription and actual high fish prescription assessed by specific scenario question [19].

The number of respondents who qualified as high fish prescribers was higher in the present study (36.2%) as compared to the study by Oh et al[19]. This finding is explained by the fact that the former study's population comprised of cardiologists whereas the latter study's population was primary care physicians. Cardiologists are expected to be better informed about recent advances in CVD prevention than primary care physicians. Moreover, the volume of CVD patients is larger in a cardiologists practice, increasing the likelihood of more frequent prescription of omega-3 fatty acids.

The low level of prescription of fish advice was present despite a strong knowledge of benefits of fish diet and a very optimistic attitude towards fish prescription. Evidently, a high knowledge and positive attitude is not translating into optimum clinical practice. This is because physician prescribing behaviors are complex [24,25]. Despite the presence of evidence based recommendations in the form of comprehensive guidelines, practice of physicians changes only modestly. Nutrition training and lack of knowledge have been recognized as barriers for physicians in studies looking at general dietary advice [26,27]. To improve clinical practice, individual predictors of prescription need to be assessed.

The importance of omega-3 fatty acid prescription lies in its effect on reduction in cardiovascular mortality [28-33]. It is the single most important dietary intervention that decreases all-cause mortality in post myocardial infarction patients. Reduction in sudden cardiac death accounts almost completely for this benefit and it is due to the anti-arrhythmic effect of omega-3 fatty acids [18,34-36]. Also, a reduction in the process of atherosclerosis has been noted [33]. Knowledge of prevention of sudden cardiac death with use of fish oil emerged as the only independent factor associated with fish oil prescription by the cardiologists when controlled for other variables. This is an interesting similarity to Oh and colleagues' findings [19]. It highlights the importance of this particular knowledge variable over other knowledge variables; it is essential not only in primary care physicians but even in the cardiac specialists. It consolidates the notion proposed by Oh and colleagues that specific knowledge of sudden death reduction may be both important and persuasive enough to improve recommendations over a general knowledge of fish benefits in CVD.

Another important factor that could have influenced the current prescription practices of Cardiologists in Karachi is the presumed high intake of fish in the study area. Karachi, being a port city has abundance of fish. Moreover, Qidwai et al found that almost 90% of a survey population in Karachi reported to have fish included in their diet atleast once a week [37]. This data is however, from a small, cross sectional study and may not be a true reflection of fish consumption in Karachi population. A perception that the population is consuming a considerable amount of fish in the diet may keep the cardiologists from emphasizing on this intervention for their patients.

Although the majority of respondents were as well informed about fish oil's benefit in reducing cardiovascular mortality and high triglycerides, as they were about prevention of sudden cardiac death, these two knowledge variables were not independently associated with high fish prescription. Similarly, nutritional training did not emerge as an independent predictor of high fish prescription.

In addition to the knowledge variable of prevention of sudden cardiac death, Oh et al reported an independent association of high fish prescription with perception of sufficient time to counsel patients [19]. This finding is not mirrored in our study. The difference in the study population is the likely reason. The small number of cardiologists serving the enormous volume of CVD patients in Pakistan has the unfortunate consequence of a very high patient to doctor ratio. It may be expected that the handful of specialists available are too overworked for dietary counseling in all patients.

The generalizability of our results may be restricted because medical training and quality of care differ considerably in other cities and villages of Pakistan. Karachi is the largest city of the country and it is a port city with an adequate availability of fish. This is important as most patients are unable to afford it in the form of supplements. A possible downside of dietary fish prescription in Karachi is that increased intake of oily fish can be associated with toxic levels of methylmercury which is known to cause myocardial infarction and neurological damage [38,39]. This negative aspect is especially important for Karachi against the backdrop of poor regulation policies on fish and water quality control. Nevertheless, the benefits of fish consumption have been seen to exceed the risks [40,41]. Eventually, the best mode of intake is supplements and must be advised if there is no financial concern [42].

## Conclusion

The level of knowledge about the benefits of omega-3 fatty acids is high and the cardiologists harbor a favorable attitude towards dispensing dietary fish advice. However, the self-reported prescription practices for omega-3 fatty acids are not in high concordance with recommendations of organizations such as the American Heart Association and National Heart Foundation of Australia, which suggests that a large proportion of patients are not getting optimal secondary CVD prevention. It is troubling because this is not at the level of primary care but at the level of the cardiologist who is an expert on CVD prevention. An important predictor of high fish prescription has been identified: the knowledge of prevention of sudden cardiac death in CVD patients. This particular life-saving property of omega-3 fatty acids should be the focus of any implemented educational strategy targeted to improve secondary CVD prevention via omega-3 fatty acid supplementation.

## Abbreviations

CVD: Cardiovascular disease; DHA: Docosahexaenoic; EPA: Eicosapentaenoic acids; PUFAs: n-3 polyunsaturated fatty acids; SD: Standard deviations.

## Competing interests

The authors declare that they have no competing interests.

## Authors' contributions

SAG and SS conceptualized the study and were involved in the study design. HMH and WFR collected the data. AMT and ZQ were involved in the data analysis and data interpretation. SAG, SS and AMT prepared the manuscript. SHD provided critical feedback and guidance and was responsible for the study's ongoing management. All authors read and approved the final manuscript.

## Pre-publication history

The pre-publication history for this paper can be accessed here:



## Supplementary Material

Additional file 1 **Questionnaire.** The questionnaire containing a total of 23 items that asked about physician practices, knowledge, and attitudes of dietary fish supplementation for patients with known CVD is attached. Click here for file
