# Supplementary material for: Secondary prevention of heart disease – knowledge among cardiologists and Ω-3 (Omega-3) fatty acid prescribing behaviors in Karachi, Pakistan
Source: BMC Cardiovasc Disord. 2009 Jan 27;9:4. doi: 10.1186/1471-2261-9-4 (PMC2640345; doi:10.1186/1471-2261-9-4)
Supplement: Additional file 1 — Questionnaire. The questionnaire containing a total of 23 items that asked about physician practices, knowledge, and attitudes of dietary fish supplementation for patients with known CVD is attached. [file 1471-2261-9-4-S1.pdf]

QUESTIONNAIRE

INSTRUCTIONS: Unless otherwise specified, please check ONE answer to each question. Pick the answer that best matches your response.

INSTITUTION ______________________________

Q1. How many patients per week do you see with known cardiovascular disease (coronary artery disease, history of myocardial infarction)?

☐ 1-5 ☐ 6-10 ☐ 11-20 ☐ 21-30 ☐ 31-40 ☐ 41-50 ☐ >50

Q2. How often do you give general dietary advice to patients with known cardiovascular disease?

☐ Almost always (>80%) ☐ Often (60-80%) ☐ Sometimes (40-59%) ☐ Not Often (20-39%) ☐ Almost never (< 20%)

Q3. How often do you discuss dietary fish intake/supplementation such as omega-3 tablets in your patients with known cardiovascular disease?

☐ Almost always (>80%) ☐ Often (60-80%) ☐ Sometimes (40-59%) ☐ Not Often (20-39%) ☐ Almost never (< 20%)

Q4. Your patient is a 65 y/o male who recently suffered from a myocardial infarction. He does not have diabetes and his LDL cholesterol is currently 79 mg/dl on medications. His blood pressure is well controlled on a beta-blocker. He asks you for a diet to reduce his chance of "another heart attack." He is not overweight. Which diet would you recommend?
YOU MAY CHOOSE MORE THAN ONE

- ☐ Lower carbohydrate, higher protein diet (e.g. Atkins, South Beach)
- ☐ Low saturated fat, high fruit and vegetable diet
- ☐ Increased intake of fatty fish meals, at least twice a week
- ☐ Low sodium diet
- ☐ None
- ☐ Other____

FOR THE QUESTIONS BELOW, YOU MAY ONLY CHOOSE ONE

Q5a. Another patient asks your opinion about a low carbohydrate, high protein diet (e.g. Atkins, South Beach). He is similar to the patient in Q5 except he is 30 lbs overweight. How do you respond to this patient?

- ☐ Strongly recommend it
- ☐ Recommend it
- ☐ No opinion
- ☐ Advise against it
- ☐ Strongly advise against it

Q5b. This same patient asks for your advice regarding dietary fish or supplementation. How do you respond?

- ☐ Strongly recommend it
- ☐ Recommend it
- ☐ No opinion
- ☐ Advise against it
- ☐ Strongly advise against it

Q6. To your knowledge, has a diet high in fruits and vegetables been proven to lower blood pressure in hypertensive patients?

- ☐ Do not know of any study
- ☐ No, studies do not support
- ☐ Unclear; controversial
- ☐ Yes, studies support

Q7. To your knowledge, has fish oil been proven to reduce cardiovascular mortality in patients with known cardiovascular disease?

- ☐ Do not know of any study
- ☐ No, studies do not support
- ☐ Unclear; controversial
- ☐ Yes, studies support

Q8. To your knowledge, have antioxidants been proven to reduce cardiovascular mortality?

- ☐ Do not know of any study
- ☐ No, studies do not support
- ☐ Unclear; controversial
- ☐ Yes, studies support

Q9. To your knowledge, has fish oil supplementation been proven to reduce high triglycerides?

- ☐ Do not know of any study
- ☐ No, studies do not support
- ☐ Unclear; controversial
- ☐ Yes, studies support

Q10. To your knowledge, has decreasing dietary sodium been proven to lower blood pressure in hypertensive patients?

- ☐ Do not know of any study
- ☐ No, studies do not support
- ☐ Unclear; controversial
- ☐ Yes, studies support

Q11. To your knowledge, has fish oil been proven to reduce sudden cardiac death?

- ☐ Do not know of any study
- ☐ No, studies do not support
- ☐ Unclear; controversial
- ☐ Yes, studies support

Q12. **Please circle the response that best describes your beliefs about nutrition.**	Strongly Disagree	Disagree	Neutral	Agree	Strongly Agree
a. Nutrition has an important part to play in the prevention of cardiovascular disease	1	2	3	4	5
b. The cardiologist has an essential role in giving dietary advice	1	2	3	4	5
c. The cardiologist has insufficient time to advise patients adequately	1	2	3	4	5
d. Advice given will impact on what people eat	1	2	3	4	5
e. Advice given will be effective in reducing cardiovascular disease	1	2	3	4	5

13. Please check all of the settings in which you have received training or formal education in dietary interventions for cardiovascular disease
☐ Medical school
☐ Residency
☐ Other____
☐ None of the above

Q14. Consider a hypothetical natural product **A**, formulated for Disease **B**. What factors would influence you to recommend product A to your patients with disease B? *Please rank in order of importance, starting with "1"for the most important factor.*
RANK (1 through 5)
___ A biological and plausible mechanism of action.
___ Guidelines from a well-respected medical organization associated with disease B.
___ Rigorous clinical research outcome data to support product A.
___ Explicit awareness of specific doses, clinical indications, and side effect profile
___ Professional and personal interest in both product A and disease B.

Please answer the following questions about yourself.

Q16. I am: ☐ Male ☐ Female
Q17. What is your age (in years)? _______
Q18. Where did you attend medical school? ☐ Pakistan ☐ USA ☐ UK ☐ Other country, please specify _________
Q19. Where did you complete your residency training? ☐ Pakistan ☐ USA ☐ UK ☐ Other country, please specify _______
Q20. How would you classify your primary practice? ☐ Office practice ☐ Hospital-based ☐ Other _____________
Q21. Are you faculty for a residency or medical school? ☐ No ☐ Full-time Faculty ☐ Part-time faculty
Q22. Have you subscribed, or read any medical journal on a regular basis? ☐Yes ☐No
If yes to Q22, which of the journals? ☐Heart ☐NEJM ☐Circulation ☐Lancet ☐JPMA ☐JCPSP ☐others, please specify ______
